# Services for older adults in rural primary care memory clinic communities and surrounding areas: a qualitative descriptive study

**DOI:** 10.1186/s12913-024-11167-w

**Published:** 2024-06-13

**Authors:** Valerie Elliot, Julie Kosteniuk, Megan E. O’Connell, Chelsie Cameron, Debra Morgan

**Affiliations:** 1 Canadian Centre for Rural and Agricultural Health, University of Saskatchewan, 104 Clinic Place, Saskatoon, Saskatchewan (SK) SK S7N 2Z4 Canada; 2 Department of Psychology, University of Saskatchewan, Saskatoon, SK Canada

**Keywords:** Rural, Older adults, Dementia, Programs, Services, Primary care, Memory clinic

## Abstract

**Background/Objectives:**

As part of a larger study, and in collaboration with rural primary health care teams, RaDAR (Rural Dementia Action Research) primary care memory clinics have evolved and continue to spread in communities across southeast Saskatchewan, Canada. This study focuses on the geographical areas of the four communities where RaDAR memory clinics were first developed and implemented and describes the services and supports available to older adults including memory clinic patients and families living in these areas. Our goal was to identify and describe existing programs and gaps, create inventories and maps, and explore the service experiences of family caregivers of people living with dementia in these rural areas.

**Methods:**

Using a qualitative descriptive design, an environmental scan of services was conducted from December 2020 to April 2021 using focus groups (*n* = 4) with health care providers/managers (*n =* 12), a secondary source (e.g., program brochures) review, and a systematic internet search targeting four RaDAR memory clinic communities and surrounding areas via community websites, online resources, and the 211 Saskatchewan service database. Data were analyzed using content analysis; findings informed semi-structured interviews with caregivers (*n =* 5) conducted from March to July 2022, which were analyzed thematically. Geographic areas explored in this study covered an area of approximately 5666 km^2^.

**Results:**

From the scan, 43 services were identified, categorized into 7 service types, and mapped by location. Seventeen services were dementia-related. Services included social/leisure activities (*n =* 14), general support/referrals (*n =* 13), transportation (*n =* 7), information/education (*n =* 4), respite (*n =* 2), in-home care (*n =* 2), and safety (*n =* 1). Service levels included local (*n =* 24), provincial (*n =* 17), and national (*n =* 2), and were offered in-person, remotely (or both) with 20 services across 4 service types offered remotely. In general, most services had no fees, involved self-referral, and providers had a range of education/training. Key interview themes reflected the need for locally available, accessible services that offer (i) individualized, flexible, needs-based approaches, (ii) in-home care and continuity of care, and (iii) both formal and informal supports. Key gaps were identified, including (i) locally accessible, available services and resources in general, (ii) dementia-related training and education for service providers, and (iii) awareness of available services. Benefits of services, consequences of gaps, and recommendations to address gaps were reported. In general, service providers and program participants were an even mix of females and males, and program content was gender neutral.

**Conclusions:**

Findings highlight a range of available services, and a number of varied service-user experiences and perspectives, in these rural areas. Key service gaps were identified, and caregivers made some specific recommendations to address these gaps. Findings underscore multiple opportunities to inform service delivery and program participation for rural and remote people living with dementia and their families.

**Supplementary Information:**

The online version contains supplementary material available at 10.1186/s12913-024-11167-w.

## Introduction

Since dementia was declared a public health priority in a 2012 report developed jointly by the World Health Organization and Alzheimer’s Disease International, the estimated number of people living with dementia globally has grown from 35.6 million [1] to more than 55 million [2]. As these numbers continue to increase, so too does the need for dementia-related programs and services that support timely diagnosis, treatment, and disease management for people living with dementia and their care partners [2]. Evidence-based guidelines identified that early diagnosis and access to services and supports are key to achieving a better quality of life for both people living with dementia and caregivers [3]. A growing body of literature has established that in rural and remote areas, services in general are lacking, and access to specialists and dementia-specific services is limited where geographical distance from existing services is an access barrier [4–6]. A recent systematic review found that compared to their urban counterparts, rural people living with dementia had higher mortality rates, fewer physician visits, more hospitalizations, more antipsychotic medication use, less home care service use, and more use of long-term care [4]. Such findings illustrate the negative effects of geographical distance for people living with dementia and their family caregivers in rural areas.

To improve dementia-related health service delivery for people living in rural and remote areas within the province of Saskatchewan, Canada, the university-based Rural Dementia Action Research (RaDAR) program was established in 1997 [7]. The province has a population of 1,098,352 in an area 651,035 km2 and a population density of 1.9 persons/km2. Proportionally more Saskatchewan people live rurally (39%) in areas with less than 10,000 people compared to Canada overall (19%) [8]. To address the need for rural dementia diagnosis and management, the RaDAR team developed an effective, evidence-based rural primary care model for dementia based on the three main domains of team-based care, decision support, and specialist-to-provider support. Since 2013, RaDAR has collaborated with rural interprofessional primary health care teams in southeast Saskatchewan to implement and adapt the model, to ensure feasibility and sustainability at the local level [9, 10].

As part of the larger study, RaDAR rural primary care memory clinics have evolved and continue to spread in multiple communities across the southeast region of Saskatchewan [9, 10]. RaDAR memory clinics offer interprofessional, collaborative, team-based care, with slightly different team compositions depending on local context and the resources available in each community. Teams are led by a family physician or nurse practitioner and include a home care nurse or social worker, occupational therapist, physiotherapist, and Alzheimer Society First Link Coordinator, and some may include a dietitian and pharmacist. One-day RaDAR memory clinics are typically held every 1–2 months in each community where two patients and their families are seen (one in the morning and one in the afternoon). The patient and family meet with memory clinic team members together as a group at the beginning of the appointment, and then individually for assessment with each professional separately. The patient, family and team then regroup to discuss the findings and recommendations for management and follow-up.

This study focuses on the geographical areas of the four communities where RaDAR memory clinics were first developed and implemented, to identify opportunities to improve programs and services for family caregivers and people living with dementia. The purpose of the study was to describe the current state of services and supports available and accessible to this population across the continuum of care, identify gaps, and explore the experiences of service-users and their recommendations for improvement.

## Methods

### Study design

To explore our study objectives, we followed a qualitative descriptive approach [11, 12] and conducted a two-phase environmental scan in and around communities with RaDAR memory clinics to identify and describe existing programs that might be used by RaDAR memory clinic patients and families, identify gaps in services, and create inventories and maps of relevant services. These data (Phase 1) informed the semi-structured telephone interviews that were subsequently conducted (Phase 2) with caregivers of people living with dementia in these areas to explore their perspectives and experiences with programs and services.

#### Phase 1 – Focus groups, secondary source review, internet search

Data were collected through focus groups with health care providers and managers in December 2020 and January 2021, a subsequent review of secondary sources provided by focus group participants (e.g., program brochures), and a systematic internet search conducted from February to April 2021.

First, four focus groups were held with a total of 12 participants who were involved with the RaDAR memory clinics and knowledgeable about programs available in the communities. The purpose of these focus groups was to identify programs and services for older adults available in RaDAR memory clinic communities, and collect data covered by the Focus Group Guide (Additional file 1) on topics such as the training and experience of program providers, program eligibility criteria, costs, referral processes, and contact information, how programs have changed in recent years in response to the presence of memory clinics, the impact of the COVID-19 pandemic on programs and services, current patient and family needs, program gaps and innovations, and recommendations to address program gaps.

Focus group participants were all female and included three primary health care managers, two primary health care facilitators, two home care nurses, two occupational/physical therapists, two social workers, and one Alzheimer Society of Saskatchewan staff. Focus groups varied in length from 60 to 83 min, were audiotaped, and transcribed verbatim by a university transcription lab. Secondary sources were identified and materials provided by focus group participants were reviewed, followed by a systematic internet search that targeted RaDAR memory clinic communities and surrounding areas via community websites, the 211 Saskatchewan® (https://sk.211.ca/) service database, and existing links to other online resources (Additional file 2).

A data extraction tool informed by Charlton and colleagues [13] was developed and used to collect and chart information on relevant programs and services. Data were collected and charted across all three sources and included a description of the program, location, program providers, eligibility criteria, referral information, cost, contact information, and website address. Program and service data were synthesized using qualitative content analysis [12–15]. Phase 1 took place between December 2020 and April 2021. Data were charted for 43 services identified across a range of settings, and data synthesis involved categorization into 7 service types informed by Stockwell-Smith and colleagues [16]. These data were then used to inform the development of a semi-structured interview guide for Phase 2.

### Phase 2 – Caregiver interviews

For the interviews, convenience sampling was used to invite people living with dementia and/or caregivers of people living with dementia via two main methods. Posters were distributed and posted in various locations within our geographical areas of interest (such as local businesses, medical clinics, municipal offices, newspapers). The Alzheimer Society of Saskatchewan distributed study information and an invitation to participate to their clients who resided in the areas of interest. Recruitment was open to both males and females however, only one male participated.

Five semi-structured telephone interviews were conducted from March to July 2022 with family caregivers of people living with dementia in and around four communities with RaDAR memory clinics. The purpose of these interviews was to gain a deeper understanding of their experience with programs and services over the last few years (since 2019), exploring topics covered by the Interview Guide (Additional file 3) such as awareness and use of services, reasons used or not used, barriers and facilitators to access and use, recommendations to improve or workaround issues, and how the COVID-19 pandemic impacted their use of programs and services. Interviews averaged 40 min in length (range 14:38 to 65:56), were audiotaped, and transcribed verbatim by a university transcription lab. Interview data were analyzed descriptively, with descriptive statistics (frequencies and proportions) for the quantitative (yes/no) data, and thematic analysis of the open-ended qualitative data using Braun and Clarke’s [17] six-phase approach which includes (1) familiarizing with data; (2) generating initial codes; (3) searching for themes; (4) reviewing themes; (5) defining and naming themes; and (6) reporting. Transcripts were initially coded manually (in Word) and then into meaningful themes using NVivo software [18]. Themes were refined iteratively by review and discussion among study authors. Exemplary quotes are used in this paper to illustrate themes and service gaps.

### Setting

This study was part of a larger ongoing study conducted with rural primary health care teams in southeast Saskatchewan to support the timely diagnosis and care management for people living with dementia and their families within their home communities, where rural primary care memory clinics had evolved in four geographical areas including Community 1 (pop. 1,076), Community 2 (pop. 10,870), Community 3 (pop. 1,110; two villages served by one RaDAR memory clinic team, pop. 332 and 778), and Community 4 (pop. 1,524). Population numbers are based on the 2021 Statistics Canada Population Census [19]. Geographic areas explored in this study covered an area of approximately 5666 km2.

### Ethics

This study was approved by the University of Saskatchewan Behavioural Research Ethics Board.

## Results

This study identified a range of services and supports across various settings for older adults in RaDAR memory clinic communities and surrounding areas, and a number of meaningful themes.

### Phase 1 – Focus groups, secondary source review, internet search

We identified 43 services that were categorized into seven service types, including: Social and Leisure Activities (*n* = 14), General Support and Referrals (*n* = 13), Transportation (*n* = 7), Information and Education (*n* = 4), Respite (*n* = 2), Home and Personal Care (*n* = 2), and Safety (*n* = 1). In Table 1, services are illustrated by community and whether available in-person (*n* = 41) or remotely (*n* = 20). Remotely delivered services were available for 3/14 Social and Leisure Activities, 12/13 General Support and Referrals, 4/4 Information and Education, and one in the Safety category that was solely a remotely offered program.

**Table 1:** Charted services by category and community

Program & Service Types (*n*)	Available remotely	Available in-person in rural communities						^i^TOTALS
		*Community 1	*Community 2	*Community 3a	*Community 3b	*Community 4	Other surrounding communities	
**Social & Leisure Activities (*n*** **= 14)** **•** senior centres, libraries, coffee clubs, activity programs	n = 3	n = 3	n = 2	n = 2	n = 2	n = 2	n = 5	N = 19
**General Support & Referrals (*n*** **= 13)** **•** counselling, relationship/behaviour management, support groups, referrals	n = 12	nil	nil	nil	nil	nil	n = 1	N = 13
**Respite (*n*** **= 2)** • in-home, overnight, day centres	nil	n = 1	n = 2	n = 1	n = 1	n = 1	n = 1	N = 7
**Home & Personal care (*n*** **= 2)** • medical or non-medical care in the home such as Home Care, house cleaning/laundry, gardening/lawn mowing, showering, meal prep	nil	n = 1	n = 2	n = 1	n = 1	n = 1	n = 1	N = 7
**Transportation (*n*** **= 7)** • such as volunteer driver programs, Handi-vans	nil	n = 2	n = 1	n = 1	n = 1	n = 2	n = 3	N = 10
**Information & Education (*n*** **= 4)** • education/information sessions, leaflets/flyers, awareness events	n = 4	nil	nil	nil	nil	nil	nil	N = 4
**Safety (*n*** **= 1)** • MedicAlert® Safely Home® - Canada-wide medical identification service	n = 1	nil	nil	nil	nil	nil	nil	N = 1
**TOTALS (*n*** **= 43)**	N = 20	N = 7	N = 7	N = 5	N = 5	N = 6	N = 11	N = 61
		N = 41						

^i^Total N’s in this column include some same service types offered in-person in multiple communities (e.g., SE Regional Public Libraries)

*Locations of rural primary care memory clinics

Programs and services included those offered at the local (*n* = 24), provincial (*n* = 17), and national (*n* = 2) levels. Seventeen (17/43) services were related to dementia, and ten (10/17) of these were provided by the Alzheimer Society. The referral process for most programs (33/43) was self-referral (*n* = 3 referrals by health care provider; *n* = 7 missing). Where reported, nearly half of all services (20/43) were provided at no cost, *n* = 9 involved a fee per service, and *n* = 5 had membership fees (*n* = 9 missing). The education background of service and program providers were mixed, where eight programs were identified only as ‘volunteer-based’, one as having a certified fitness instructor, and the remaining included teams comprised of different combinations of health care providers from multiple disciplines such as specialists, registered and nurse practitioners, nurses, therapies, social workers, counselors, and a dietitian.

All four geographical locations of interest (communities with RaDAR memory clinics) had more than one Social and Leisure program, offered a Transportation service, Respite, and Home and Personal Care. Most services identified were in the Social and Leisure Activities category (14/43), which consisted mainly of in-person drop-in ‘senior’ centers or regional libraries that existed in all locations of interest. In this category, one ‘active living’ program evolved as a direct result of the RaDAR memory clinics. Almost all (12/13) General Support and Referrals were available remotely, and most were offered provincially (and one nationally) by a mix of publicly funded, non-profit, and for-profit organizations. One of these programs was implemented in direct response to the COVID-19 pandemic. Transportation services (*n* = 7) were offered at the local level in all communities, and just one was solely volunteer-based and available without a cost. All (4/4) Information and Education services were provided by the Alzheimer Society and were available virtually. We identified two respite programs (one publicly funded available in all communities, and one private for-profit available in the larger center) and two home and personal care programs (one publicly funded available in all communities, and one private for-profit in the larger center). Lastly, the Safety category included one privately offered national program.

### Phase 2 – Caregiver interviews

Telephone interviews with five family caregivers of people living with dementia were conducted. Four caregivers were female, and the mean age of caregivers was 70 years (range 61–82). Three family members were spouses and two were adult children. Most caregivers lived in the community with their spouse (4/5) and one person living with dementia was living in long-term care. Two of the five family members had previously attended a RaDAR memory clinic, and two had previously received a dementia-related diagnosis (Table 2).

**Table 2:** Caregiver interview participant characteristics

	N = 5
**CG Sex**	
Female	4/5
Male	1/5
**CG Age** mean (SD, range)	70 (10.1, 61–82)
**CG Relationship to CR**	
Spouse	3/5
Adult Child	2/5
**CR Sex**	
Female	3/5
Male	2/5
**CR living arrangement**	
In community with spouse	4/5
In LTC with spouse	1/5
**Attended a rural primary care memory clinic**	
Yes	2/5
No	3/5
**CR Received dementia-related diagnosis**	
Yes	2/5
No	3/5

CG = caregiver; CR = care recipient; LTC = long-term care

Across the seven service types (Table 3), all caregivers reported being aware of at least some sort of social and leisure activities, and services for respite, home and personal care, and safety. Most (4/5) were also aware of some type of general support and referrals, education/training/information, and (3/5) knew about transportation services. Caregivers reported that either they or their family member living with dementia had used mostly home and personal care services (4/5), and social and leisure activities (4/5). Safety services and general support and referrals were used by 3/5, respite and education/training/information by 2/5, and zero reported use of a transportation service. All but one of the services used were perceived to be a helpful service. There were mixed reports across various service types of features that made a service easier to access and use, as well as features that made a service difficult to access or use, potential ways to work around those difficulties, and other recommendations. Where reported, service providers and participants were an even mix of females and males, and program content was perceived as primarily gender neutral (Table 3). Lastly, caregivers identified three services that were not captured in Phase 1 (one general support and referrals, and two social and leisure activities).

**Table 3:** Frequencies of caregiver interview (*N* = 5) responses to yes/no questions by service category

Response	Service Category						
Yes	General Support & Referrals	Respite	Home & Personal Care	Transport-ation	Education, Training, Information	Social & Leisure Activities	Safety
Aware of	4/5	5/5	5/5	3/5	4/5	5/5	5/5
Used	3/5	2/5	4/5	0/3	2/4	4/5	3/5
Group service	1/3	0/2	0/4	n/a	1/2	4/4	0/3
Female provider	3/3	1/2	2/4	n/a	1/2	2/4	0/3
Impact of provider sex	0/3	0/1	0/2	n/a	0/1	0/2	0/3
Content gender-neutral	0/3	0/3	0/3	n/a	0/3	2/3	0/3
Helpful service	3/3	1/2	4/4	n/a	2/2	4/4	3/3
Recommendations	3/3	0/2	2/4	n/a	1/2	3/4	0/3
Difficulty	2/3	1/2	1/4	n/a	1/2	2/4	1/3
Workaround	2/2	0/1	1/1	n/a	1/2	2/4	1/1
Made easier	3/3	1/2	2/4	n/a	2/2	4/4	1/3

#### Key themes

Key themes reflected the need for locally available, accessible services that offer (i) individualized, needs-based approaches, (ii) in-home care options and continuity of care, and (iii) both formal and informal supports. The frequencies of emerged themes across the five interviews are shown in Table 4.

**Table 4:** Frequencies of emerged themes in caregiver interviews (*N* = 5)

Caregiver	Participant 1	Participant 2	Participant 3	Participant 4	Participant 5
Theme					
Individualized, flexible, needs-based approach	✓	✓	✓	✓	✓
Continuity of care in-home care options	✓		✓	✓	✓
Both formal and informal supports	✓	✓	✓		✓

#### Individualized, flexible, needs-based approaches

All caregivers described the importance of programs and services using a person-centred approach to care that is based on their individual needs and circumstances. Each person living with dementia and their respective family caregivers have unique situations and needs that require different care, supports, and information. Examples included differences in disease stage or trajectory, health literacy level, technological capability, mobility, community resources, or any number of individual or contextual circumstances or situations. Providing resources that are appropriate, relevant, understandable, and easily accessible and useable was a notable concern.

*The one thing that I might say is that they create programs that are kind of cookie cutter. I would [suggest that] during those stages, that there’s links made to services and supports that coincide with those stages. And to acknowledge and understand the different circumstances which people live, and the supports and service they might need. Like home care, it can’t look the same in every community, because every community’s needs are different.* (CG1)

Flexible scheduling would be helpful for caregivers who work outside the home, even when services are provided virtually.

*And me, with my work schedule, when they have [online exercise program] and all that, it’s when I’m working. So I can’t do it with him […]. My work and their schedule doesn’t coincide […]. And with technology […] he needs me.* (CG2)

#### In-home care and continuity of care

Almost all (4/5) caregivers reported using in-home care services. In particular, home care was said to be an excellent provider of personal, in-home support services, and provided information about other programs and services available in their community.

Although one caregiver reported their family member living with dementia was resistant to anyone providing care other than herself, in general most reported being very amenable to home care and happy with their experience. Local services and providers were generally perceived as familiar and providing more personalized care. Home care appeared to benefit both caregivers and people living with dementia. For example, caregivers were typically not required to be present during home care visits which allowed them time for a short break or to run errands.

*I wasn’t always here when they were here, I could go out if necessary […]. We actually look forward to them coming […]. Oral and tooth care, and even shaving […] it was very helpful because I’m not sure that he would be in favour of me helping him in regards to – I did bath him a few times too, but the other care, he was quite pleased with someone else besides me came to care for him.* (CG3)

Home care facilitated connections to other valuable programs and services such as assessments, respite, the Alzheimer Society, or even housekeeping.

*“Our first contact [with Alzheimer Society of Saskatchewan] was through home care, because we were ground zero. So we were trying to figure out what the process would be. And so, home care actually had given us some options, and we had in-home care for my dad who had different issues. But my mom was always kind of at the first and foremost of our thoughts on, how are we going to get the ball rolling to have her assessed. And then, you know, look at the options for care for her … that [assessment; respite] was actually all arranged through home care.* (CG5)”.

Continuity of care was identified as a key concern among caregivers where consistency in services and providers could be helpful.

*Depending on who was the [in-home care] person that came, the service [they provided] was different. Three of four of the folks that came in the morning were excellent. They actually washed her face and got her up and going if she wasn’t up. And the other one, all she did was offer her her medication and that was it.* (CG4)

#### Formal and informal supports

The importance of having both formal (agencies, institutions, professionals) and informal (family, friends, community) supports was noted by most (4/5) caregivers. In addition to health-related programs and services such as home care or respite, examples of formal supports included support groups, social programs (mainly provided by the Alzheimer Society), and transportation services.

*[re: transportation services] Because he has no driver’s license anymore, well that will help him to go to see his friends or his brother, or something like that, that are not at walking distance.* (CG2)

A lack of formal supports was at times offset by informal supports.

*[re: support groups] I haven’t accessed any of that. Just because I’m pretty fortunate, I’ve got a really good core group of family and friends, that I haven’t really felt that I’ve needed that kind of support, so I’m pretty fortunate there.* (CG1)

*[re: safety] The neighbours would kind of look out for them as well. So, that was helpful.* (CG5)

#### Key gaps, benefits, and consequences

Several gaps in programs and services were identified, including (i) locally accessible, available services and resources in general, (ii) dementia-related training and education for service providers, and (iii) awareness of available services.

#### Locally accessible, available services and resources

The main gap identified was a lack of services and supports in general, reported by all caregivers, noted as particularly worse for very small communities, and during the pandemic.

*And it was really difficult in rural areas to find a housekeeper to come in and do light duties that isn’t already swamped.* (CG1)

Even though local services and providers in the smaller centers offered a sense of familiarity and comfort, relocating to a larger center was at times necessary.

*She’s actually moving from the little town where she did get a bed, into the [larger center]. So she’ll be in there and she should have people that are trained to deal with her day-to-day life as she goes on now. But she has had terrific care in that little [care] home in [small center], second to none actually in terms of the care they can offer her. So we’re anxious to try that and see how that’s going to work […]. So she broke her hip a month ago and there’s no physio at [smaller center]; it’s also closer for us. But it’s not about us it’s about her.* (CG4)

*And it’s very small town, so knew most of the staff previously, and so that gave me a lot of comfort too.* (CG5)

Although the pandemic negatively impacted most services and supports and increased isolation, there were also positive aspects mentioned such as the emergence of new service delivery options such as grocery delivery or curbside pickup, and virtual online access to programs.

*A total godsend, is the local [grocery store] has been doing grocery delivery. And that service was available before, but it wasn’t necessarily easy access or always available. Like, they’d only maybe have somebody delivering on Tuesdays and Thursdays, and whatever. But now it is available with a phone call. It’s available by fax. It’s available over the internet. And it’s now there when the store is open, if you make your call in by 10 o’clock in the morning, you’re gonna get delivery that day. And everybody knows about it. And things like drugstores […] you can go online, you can literally look at their products in a picture book, you can select what you want, set up a time that you’re gonna be there, pick it up, and they walk it out to you. You can pay with your card and they literally walk it out and put it in your trunk, and you drive away. Those kinds of services that make for ease of accessibility have been great.* (CG1)

However, the complexities involved with options such as online grocery orders or virtual programming rendered them not feasible for everyone.

*How many seniors do you know that knows how to use a computer? Not many. So it’s [the program/service] not accessible. That’s the thing. They are there, but you can’t use them. Too new. It’s a new tool, and they never learned it.* (CG2)

Consequences of existing gaps in locally accessible, available services and resources included isolation, delayed assessment, and care crises.

*Every time I phone, I get the answering machine, they didn’t call back. So I’m like, well, I don’t know what’s going on there.* (CG2)

*The doctoring definitely suffered, because the doctor wasn’t seeing them at all, in-person. So there’s another reason why she wasn’t assessed. Because he couldn’t even see her. He wasn’t taking patients in-person. So, he was just doing phone interviews. When they did try to have an assessment with her, it was- They were unable to do it because she was too far advanced and wasn’t really agreeable to it.* (CG5)

*I had to call the ambulance about three days ago, in the night, because of his breathing, and that, but he’s some better. I can’t see that he would be able to come home now. He’s totally bed-ridden and he needs help.* (CG3)

#### Dementia-related training and education for service providers

There appeared to be a gap in dementia training and education among service providers, particularly noted in smaller communities. This could lead to a lack of caregiver confidence in service providers, and perhaps relocating to a larger centre.

*She’s [spouse living with dementia] been volunteering at the home for years and she’s looked after dementia patients in her family. She’s pretty well cased on it. A lot better than a lot of the nurses are that are at our hospital.* (CG4)

#### Awareness of available services

There were gaps in the awareness of existing available programs and services. In general, most caregivers were aware of (or using) Alzheimer Society services, however there did seem to be less awareness of other available programs and services. Health care providers were often reportedly unaware of what relevant services were available. Other than the Alzheimer Society, home care and personal connections appeared to be the main link to awareness of, and how to connect with, other services.

*We were looking and we got a list of names of housekeepers from the hospital. But none of those people that we had originally called were available or had room. And it just happened that mom’s neighbour […] we phoned her, and she came and met with Mom and I. And we talked about what we might need, and she agreed to take Mom on. Yep. Follow-up [by the hospital] after would’ve been helpful, because some of the people’s names who were on the list, they had retired. And one of the ladies said to me, “Well, I’m really not sure why my name’s still on there. I haven’t done that for three years.” And I said, “Well, I’m sorry.” I did call back and I did mention to [hospital social worker] at that time, that that person was no longer actively doing that. So that they were aware. Because it was probably something that somebody had retired and just not bothered to tell somebody. […] It’s almost like you need somebody to be a clearing house within the system, who every now and then just double-checks that those services and those contact numbers, especially when they’re not housed underneath their umbrella, right, to ensure that those things are still available.* (CG1)

A lack of awareness about relevant, available services was problematic and led to unnecessary difficulty with making those connections.

#### Caregiver recommendations to address gaps

Caregivers made recommendations to increase the number of dementia-related programs with a wider range of schedule (dates/times) and format options to allow for greater flexibility and increase access for those with multiple commitments and competing schedules. Virtual options enhanced access for many but not for those without the technology or the ability to use it. It was also suggested that more dementia-related training be given to program and service providers to improve their understanding of dementia and provide them with effective approaches to improve the receptiveness and participation of people living with dementia.

The recommendation was made to offer some dementia stage-specific programs and services that would better meet the personal needs of individuals and caregivers currently experiencing specific stages of dementia. A further recommendation was to provide information that links services and support to certain dementia stages. Caregivers also reported on the importance of clinics, hospitals, and service providers maintaining current, up to date lists with information about available relevant services and supports in both the private and non-private sectors. This would ameliorate the time-intensive process of individuals and caregivers doing their own extensive search. Caregivers reported that the emergence of the RaDAR memory clinics facilitated awareness of dementia-related programs and services and making those connections. Lastly, caregivers made recommendations for consistency of care in terms of having the same care providers and providing care in the same way, and further, that service providers maintain their connection to people living with dementia and their caregivers so that changing needs can be addressed as they happen.

## Discussion

This study adds to the literature by mapping existing services and supports and identifying opportunities to improve service delivery and interventions in rural and remote communities for people living with dementia and family caregivers. Although programs, services, and supports were lacking in general, particularly in smaller communities, we identified and described a range of relevant programs and services that were available to RaDAR memory clinic patients and families. In phase two, we deepened our understanding of the experiences of service-users, and identified key themes, gaps, benefits, consequences, and recommendations to address gaps. We found that services that offered (i) individualized, flexible, needs-based approaches, (ii) in-home care and continuity of care, and (iii) both formal and informal supports were important to caregivers who described the benefits of same. We found key gaps including (i) locally accessible, available services and resources in general, (ii) dementia-related training and education for service providers, and (iii) awareness of available services, and the consequences of these gaps, along with recommendations to address. Overall, these findings align with those reported in a recent scoping review on the personal experiences of rural people with dementia and their caregivers, and reflect multiple challenges associated with rural dementia care services and opportunities for improvement [20].

In this study, seventeen services were related to dementia, which can mainly be attributed to the strong presence of the Alzheimer Society which provided more than half of these services. This could reflect the growing need for rural dementia services and the Alzheimer Society’s work to address that service gap [5, 21–26]. Although we found that many in-person programs and services were halted during the COVID-19 pandemic, new remotely offered services emerged. Others have also reported that remotely offered strategies and services evolved or became more prevalent during the pandemic shift away from in-person services [27, 28].

Service delivery was available remotely for almost half of all services, with all but one general support and referrals, the safety program, and all education and information, provided remotely. Although caregivers reported that remotely available services improved access for some, there were technological capabilities that limited access for others. Similar benefits and challenges with technology have been reported for rural adults in general [29], and specifically for people living with dementia [30, 31]. Benefits included having access to programs and services from their own home that would otherwise require travel to a larger center, and challenges such as low internet connectivity or finding the technology too complex to use on their own, at times even with assistance [30, 31]. However, as the population continues to age globally [32], technology will advance, and so too will the related capabilities of older generations, so the importance of remotely delivered services for this population cannot be understated.

We found that a flexible, person-centered approach was necessary to fully meet the needs of different individuals. Caregivers described the importance of having access to services that offer a variety of schedules and formats to give service-users options to select what worked for them, in their individual circumstances. The importance of rural services for people living with dementia using an individualized, tailored approach is a key finding reported in a recent review [20]. Common features of dementia-related person-centered care include a holistic, respectful and dignified approach with an active partnership between health care providers, patients, and caregivers; joint information and decision-making; and individualized care tailored to meet each person’s unique needs and preferences and provide opportunities for meaningful engagement [33]. Co-designing services for and with people living with dementia is becoming increasingly acknowledged in the literature as an essential collaborative component in the development of dementia-related services [34].

Four types of services were available in-person across all communities in this study, namely social and leisure activities, transportation, respite, and in-home care services. The common presence of local regional libraries and senior drop-in centres in these communities could explain this finding in part, where most services overall fell into the social and leisure activities category. However, the recent ‘active living’ program that evolved as a direct result of the RaDAR memory clinics suggests that there remains a need for more services like these. The importance of social interaction for people living with dementia and their carers cannot be overstated and is widely supported across the literature [Roberts et al. 2023].

Although we found that some sort of transportation service existed in all RaDAR memory clinic communities, most but not all caregivers were aware of these, and none reported having used them. The negative effects of health-related stigma as it pertains to service use is well-documented in the literature [35–37], particularly for people living with dementia [20, 38, 39] or other neurological conditions [40], and their carers, which could be a contributing factor to our findings of an absence of transportation service use. We found that caregivers and their family member living with dementia often did not feel like they needed a transportation service, and preferred using more informal services (e.g., being driven by a family member). A reliance on, or preference for, informal supports (such as friends or family) has been reported elsewhere in the literature for rural people living with dementia and their carers where geographical distance is a service access barrier [20, 38]. This could also explain our similar findings regarding respite, where although respite and home care were provided by the provincial health authority across all communities, and all caregivers were aware of these services, respite was used by less than half of the caregivers we interviewed. The rationale for not using respite included a resistance on their part, or on the part of their family member living with dementia, to receive support from anyone they were not very familiar and comfortable with, particularly in the absence of their family caregiver.

In contrast, home care services were used by all caregivers in this study, were highly valued, and played a significant role in providing in-home care, and in connecting clients to other relevant, available, formal (such as therapies, memory clinic, Alzheimer Society) and informal (such as housecleaning, grocery delivery, chat groups) services and supports. This could be explained by the knowledge, expertise, and familiarity of home care providers with the community, existing services, and how to connect with those services [41]. The importance of providing in-home care for rural people living with dementia and their carers has been reported by others and has been cited as a key factor in addressing service accessibility issues for this population [25].

Although caregivers in this study preferred receiving services from more familiar providers close to home, gaps in services and access barriers contributed to consequences such as increased isolation, delayed assessment, treatment, and care crises that could result in moving to a larger center with more services. Roberts and colleagues 2023 review [20] found similar feelings of familiarity and comfort with local services and providers were reported across several studies, as were similar challenges with dementia services that negatively impacted their care including difficulties with obtaining a diagnosis, complex care systems, and a lack of services, supports, and continuity of care. This study builds upon those findings and offers recommendations for improvements that include delivering more services in general, and specifically more person-centred services, and increasing education and awareness for both service providers and the public. Caregivers in this study recommended that services be offered in a variety of formats (such as in-person and virtual), with a wider range of schedules to better serve and accommodate more people. They further recommended that available services could be mapped to the different stages of dementia, so people could more easily identify which services might be the most appropriate or relevant for them at any given stage or point in time, and that resources be easy to understand, accessible and useable. These are specific caregiver recommendations that exemplify the unique, diverse needs of this population, and the importance of offering more individualized service options.

We also found a perceived gap in dementia-related education and training among service providers. Caregivers suggested that more dementia-specific training for program and service providers was needed to increase their understanding of dementia, thereby improving their capacity to deliver, and provide connections to, appropriate services that reflect the individual needs of persons living with dementia and their caregivers. Findings like these have been reported elsewhere where a lack of dementia training and awareness of other relevant services available was a barrier to accessing supports for people living with dementia and their families [38].

Lastly, this study found that sex and gender did not play a significant role in programs and services in general, where service providers and program participants were reported as an even mix of females and males, and program content was typically gender neutral. It is possible however that our lack of findings may have been impacted by our small sample size. A recent narrative review on gender issues in the care of the elderly found that use of a gendered lens is vital to effectively meet the health needs of older adults due to the differential effects of gender on aging and illness, and points toward the role of gender as a determinant of health for the older population [42]. The importance of considering sex and gender is exemplified in recent health research in general [43–45], and specifically as it pertains to dementia [46] and rural community-based services [24], where it has been reported as lacking [47].

### Strengths and limitations

A limitation of this study is that although we used a systematic approach to identifying existing programs and services, our list is not exhaustive. It is possible that some were missed due in part to the search strategy used, and partly due to the constantly changing nature of programs and services, further exacerbated by the COVID-19 pandemic. Study authors acknowledge that having used what is essentially an unreproducible search engine that is subject to variability across users and time for the internet search in phase one is a limitation of this study. Phase one data collection for the researchers was negatively impacted by the pandemic. Many programs and services were on hold (in particular, in-person) or modified (for example offering remote participation options). Along that same vein, from the researchers’ perspective, recruitment for the caregiver interviews in phase two may have also been negatively affected (low number of participants). In addition, although this study intended to interview both family caregivers and people living with dementia, only caregivers were successfully recruited. Future research should aim to include the voice of rural people living with dementia.

The qualitative descriptive approach was a study strength that allowed for a deeper understanding of unmet service needs and access barriers by including the voices of family caregivers of people living with dementia. This was key to gaining their insight and perspective, and highlighting their experiences with services, their needs, concerns, and recommendations to improve. We have gathered a rich set of data that might help inform future research and policy in this geographic region. However, our sample was small and the geographical area of interest may limit the generalizability of our findings.

## Conclusion

This study found a range of services available for older adults in rural memory clinic communities, and provides insights into experiences with using services in these geographical areas. The current study identified important aspects of services, and the relative benefits, along with key service gaps, and recommendations to address these gaps. Findings emphasize varied challenges related to service participation faced by rural and remote people living with dementia and their families, and point to multiple opportunities to inform changes to service and program delivery.

### Electronic supplementary material

Below is the link to the electronic supplementary material.

**Supplementary Material 1: Additional File 1**. Focus group guide. This semi-structured guide was used for each of the four focus groups conducted with health care providers and managers.

**Supplementary Material 2: Additional File 2**. Secondary source review, internet search, and larger charted data file. This file includes the larger charted data file from which Table 1 was drawn, a description of the secondary source review, and the internet search process.

**Supplementary Material 3: Additional File 3**. Caregiver Interview Guide. This semi-structured interview guide was developed based on Phase 1 findings, and used for each of the five caregiver interviews.

## Data Availability

The Phase 1 dataset of this article is included within the article and its additional file (Additional file 2). The Phase 2 dataset generated and analyzed during the current study are not publicly available for reasons of participant confidentiality due to small sample size.

## Abbreviations

RaDAR: Rural Dementia Action Research Team
