# Supplementary material for: Services for older adults in rural primary care memory clinic communities and surrounding areas: a qualitative descriptive study
Source: BMC Health Serv Res. 2024 Jun 13;24:725. doi: 10.1186/s12913-024-11167-w (PMC11170901; doi:10.1186/s12913-024-11167-w)
Supplement: Supplementary file 1 — Supplementary Material 1: Additional File 1. Focus group guide. This semi-structured guide was used for each of the four focus groups conducted with health care providers and managers. [file 12913_2024_11167_MOESM1_ESM.pdf]

Environmental Scan of Community Programs

Focus Group Guide

Introduction

The objectives of this focus group are to help us to better understand the local community programs that are currently providing post-diagnostic services to clients, such as RaDAR memory clinic patients and families.

1. Can you tell us about the local community programs that you are aware of that provide post-diagnostic services to individuals who have been diagnosed with dementia? We are particularly interested in the communities where RaDAR memory clinics are available, or communities where memory clinic patients would travel for services.

2. Are you aware of the training and experience of these local community program providers?

3. How do you think local community programs have changed in recent years in response to the **presence of the RaDAR memory clinics**?

4. Have there been recent improvements in local community programs that provide post-diagnostic services to individuals with dementia and their families? (general)

5. How would you describe current needs for local community programs that provide post-diagnostic services to individuals with dementia and their families?

6. How would you describe gaps in local community programs that provide post-diagnostic services to individuals with dementia and their families?

7. Would you have any recommendations to address gaps in local community programs that provide post-diagnostic services to individuals with dementia and their families?
