# Supplementary material for: Services for older adults in rural primary care memory clinic communities and surrounding areas: a qualitative descriptive study
Source: BMC Health Serv Res. 2024 Jun 13;24:725. doi: 10.1186/s12913-024-11167-w (PMC11170901; doi:10.1186/s12913-024-11167-w)
Supplement: Supplementary file 2 — Supplementary Material 2: Additional File 2. Secondary source review, internet search, and larger charted data file. This file includes the larger charted data file from which Table 1 was drawn, a description of the secondary source review, and the internet search process. [file 12913_2024_11167_MOESM2_ESM.pdf]

Additional File 2. Secondary source review, internet search process, and larger charted data file

Charted service data file *- the larger data set working file from which Table 1 was drawn (Table 1. Charted services by category and community)*

- all programs and services identified in Phase 1 (from all sources – focus groups transcripts and secondary sources [n=3 pamphlets], and internet search) in our locations of interest (four rural primary care memory clinic communities and four small surrounding communities identified by focus group participants)

Internet search

- **Targeted searches** of community municipal websites for each respective locale (***no supplementary information was obtained***).
  - o Google searched each location of interest for a municipal website (e.g., Town of Xxxxx) and when identified, the website was then manually searched for the existence of relevant programs and services.
- **211 Saskatchewan (service database)** https://sk.211.ca/
  - o search criteria included the name of the community, and "Older Adults", for each location of interest.
  - o Data was then charted and supplemented with additional program information on public websites where available.

Table notations

- Programs identified in **211 search only** (not identified in focus groups) are **not asterisked.**
- Programs identified in **focus groups only** (not identified in this 211 search) are currently **asterisked** *.
- Programs identified **in both the 211 search and the focus groups** are currently **double-asterisked** **.
- Programs **from outside our locations of interest that focus on care for rural areas** and offer (some) remote services identified and added post-hoc are noted with a green/yellow asterisk *.

Community Program	Location (Town)	Objectives	Procedures	Eligibility criteria	Referral process	Cost	Other
****Sask Health Region Patient Healthline 8-1-1 Provided by: Saskatchewan Health Authority**	Province-wide<br><br>**Provincial**	Offers professional health or mental health and addictions advice, education and support.	Confidential, 24-hour health and mental health and addictions advice, education and support telephone line available to the people of Saskatchewan.	Saskatchewan residents.	Self-referral	Free	**Education/Training of Program Providers:** experienced and specially trained Registered Nurses, Registered Psychiatric Nurses, and Registered Social Workers

* **Bridging the Distance Provided by: Envision Counseling and Support Centre Inc.**	Community 2, Community 4<br><br>**Community-based**	*Bridging the Distance* is a program that specifically **offers support and connection** for individuals (or their family members) age 55+ experiencing isolation in Southeast Saskatchewan	Voluntary phone connection for 55+<br><br>Sessions happen in the comfort of your own home via telephone or video chat and are approximately one hour with a registered counsellor. The client guides the conversation and talks about any issues or challenges they may be facing due to isolation, loneliness, grief, etc. Privacy and confidentiality are of utmost importance and will always be taken into account.<br><br>When you call the nearest Envision Office, ask to be referred to the *Bridging the Distance* program. An Intake Counsellor will then contact you within 1-2 business days. The Intake Counsellor will answer any questions you may have, learn about you and your situation and book an appointment that works for you. A registered counsellor will then call you at your scheduled appointment	Age 55+, people living with disabilities, or housebound individuals (or their family members or other individuals), in Southeast Saskatchewan.	Self, other	Free	Implemented in direct response to the pandemic.<br><br>**Education/Training of Program Providers:** Registered Counselors

			time. *Bridging the Distance* is a voluntary program and participants are welcome to end services whenever they wish.<br>If a family member or individual would like **counselling, or just to talk**, they can call the office to connect with a counsellor. This confidential program can be accessed from the comfort of your own home via telephone or video chat.				
* **Friendly Phone Program**<br>**Provided by:**<br>**Red Cross Canada**	**National**	To help keep vulnerable seniors in their home longer by reducing isolation and increasing their health and safety.	Provides socially isolated seniors with social interaction through volunteer phone calls once a week, along with potential referral resources based on needs.	Community-dwelling, age 55+ who identify as vulnerable and who would benefit from social inclusion services. Potential clients undergo an intake interview phone assessment by Friendly Phone Coordinator and suitably paired with volunteer.	• Self, family, or caregiver<br>• Home Care or Social Services<br>• Church/Clergy<br>• Seniors' Club/Group	Free	Pre-pandemic, would go right into the home and/or by phone;<br><br>**Education/Training of Program Providers:** Volunteers are screened and educated in Red Cross confidentiality and privacy. Policies are in place re: client safety and/or complaints. Red Cross has consulted with Health Regions to ensure best practices.

**Dementia Helpline Provided by: ASOS**	Province-wide<br><br>**Provincial**	"Learn More Live Well"<br><br>The Alzheimer Society of Saskatchewan's programs and services help people with dementia, their care partners, families, and friends by providing information, support, education and referral to other community services.	Call to ask questions about dementia	People living with dementia and/or caregivers of people living with dementia	Self, other	Free	**Education/Training of Program Providers:** Not identified
****First Link® Provided by: ASOS**	Province-wide<br><br>**Provincial**	"Learn More Live Well"<br><br>First Link is about bringing together Alzheimer Society staff, primary care physicians and other health professionals to make it easier for people with dementia and their families to get the help they need.	First Link® focuses on service coordination, resources and planning, clients become more confident and involved in their own care, reducing the burden on family physicians, long-term care and emergency services. Families also are better equipped to create their own action plans to tackle future legal, financial and health needs.	People living with dementia and/or caregivers of people living with dementia	Self, healthcare provider, other	Free	**Education/Training of Program Providers:** Not identified
****Support Groups**<br>• **<u>Multiple</u> support groups offered**<br>**Provided by: ASOS**	Province-wide<br><br>Currently being offered virtually due to the pandemic.	"Learn More Live Well"<br><br>Our Support Groups for people with dementia and their caregivers provide a safe environment where you can learn, laugh and help each other through mutual understanding.	Alzheimer Society support groups offer a chance to:<br>• Exchange information and friendship with others living with and affected by dementia<br>• Access the most current information	People living with dementia and/or caregivers of people living with dementia.<br><br>Various support	Self, other	Free (Pre-registration required)	**Education/Training of Service Providers:** None identified

	**Provincial**		• Learn and share practical tips for coping with change<br>• Decrease feelings of loneliness and isolation<br>• Express feelings and be reassured that these feelings are normal<br>• Find a sense of hope	groups offered for specific subgroups such as early stage, FTD, young carers			
* **Behavioural Expressions Program**<br><br>**Provided by: Geriatric Services Out-patient Clinic (Southern Saskatchewan)**<br><br>**Saskatchewan Health Authority**	Southern Saskatchewan<br><br>**Provincial**	In addition to other services geared for older adults, with the goal of leading efforts to ensure the older person in southern Saskatchewan receives care to:<br>• Improve quality of life<br>• Optimize independence, autonomy, health and safety in the community<br>• Work with the adult senior and family members to ensure their preferences are maximized<br>• Prevent and avoid unnecessary emergency department visits and hospitalizations.<br>The Geriatric Services Resource Team will work with caregivers, Primary Care Providers,	• Comprehensive Behavioural Assessment<br>• Consultation with Interdisciplinary Team<br>• Comprehensive Recommendations including medications, environment, care provision, approach, etc.<br>• Remote follow-up as needed<br>• Admission to Dementia Assessment Unit<br>• Enhanced Observation	Anyone residing in Southern Saskatchewan with symptoms of behavioural expressions (verbal or physical) of which are either causing a safety concern or impacting patient care needs	Referrals are accepted, with clients' consent, from:<br>• Primary Care Physicians<br>• Nurse Practitioners	Free	**Education/Training of Service Providers:**<br>The Geriatric Services Resource Team is comprised of the following health care professionals:<br>• Geriatric Psychiatrist<br>• Family Physician with interest in geriatrics<br>• Nurse Practitioner<br>• Occupational Therapist<br>• Pharmacist<br>• Continuing Care Consultants<br>• Social Worker<br>• P.I.E.C.E.S.TM Manager<br>The health care professional that is involved with the client will depend on the identified needs.

		Health Care Professionals and Community Support Services.					
* **Virtual Rural and Remote Memory Clinic (vRRMC) Provided by: Rural and Remote Memory Clinic, Saskatchewan Health Authority**	Rural Saskatchewan<br><br>**Provincial**	To increase the availability and accessibility of dementia care in rural and remote areas, the clinic streamlines assessment and diagnosis in order to reduce repeated travel over long distances and to shorten the time to diagnosis by coordinating an interdisciplinary assessment on one day.	Multi-disciplinary team one-day specialist clinical assessment and follow-up to report diagnosis suggest management plan. Remotely-delivered dementia-related interventions (RRMCi) are also offered to clients and families.	Focus on atypical and complex cases of suspected dementia	Referral required from Saskatchewan Health Care Provider (physician, nurse practitioner, nurse, allied health professionals)	Free	**Education/Training of Service Providers:**<br>•Neurologist<br>•Neuropsychologist<br>•Clinic Nurse<br>•Psychometrist<br>•Physiotherapist
* **Rural and Remote Memory Clinic 2.0 (RRMC2.0) Provided by: Rural and Remote Memory Clinic, Saskatchewan Health Authority**	Rural Saskatchewan<br><br>**Provincial**	To increase the availability and accessibility of dementia care in rural and remote areas, the clinic streamlines assessment and diagnosis in order to reduce repeated travel over long distances and to shorten the time to diagnosis by coordinating an interdisciplinary assessment on one day.	Remote-delivered memory assessment by Neuropsychology team (over the telephone) with report information sent back to family doctor in order for team to remotely assist family doctor to make a diagnosis of dementia if supported by finding of the assessment and to help facilitate supports for participant. Remotely-delivered dementia-related interventions (RRMCi) are also offered to clients and families.	Focus on atypical and complex cases of suspected dementia	• Self-referral, family-member referral, referral from Saskatchewan Health Care provider, or direct referral from Alzheimer Society of Saskatchewan	Free	

* **Sleep Intervention, Rural and Remote Memory Clinic Interventions (RRMCi) Provided by: Rural and Remote Memory Clinic, Saskatchewan Health Authority**	Rural Saskatchewan<br><br>**Provincial**	To increase the availability and accessibility of a dementia-related sleep intervention, to increase the quality and quantity of restful sleep.	A remotely-delivered sleep intervention designed to help older adults with cognitive concerns of dementia.	Available to adults with cognitive concerns/dementia, and caregivers of persons with mild cognitive impairment or dementia.	• Self-referral, or referral from a health care provider	Free	Delivered by PhD in psychology with assistance from graduate-level psychology students.
* **Cognitive Rehabilitation, Rural and Remote Memory Clinic Interventions (RRMCi) Provided by: Rural and Remote Memory Clinic, Saskatchewan Health Authority**	Rural Saskatchewan<br><br>**Provincial**	To increase the availability and accessibility of a dementia-related cognitive rehabilitation intervention.	Individually-tailored, remotely-delivered, and empirically-based intervention rooted in the principles of cognitive rehabilitation.	Available to adults with cognitive concerns/dementia, and caregivers of persons with mild cognitive impairment or dementia.	• Self-referral, or referral from a health care provider	Free	Delivered by PhD in psychology with assistance from graduate-level psychology students.
* **Virtual Socialization Groups, Rural and Remote Memory Clinic Interventions (RRMCi) Provided by: Rural and Remote Memory Clinic, Saskatchewan Health Authority**	Rural Saskatchewan<br><br>**Provincial**	Virtual socialization groups to address COVID-19-related loss of social interactions and foster a sense of belonging for older adults in Saskatchewan with groups of people who have common interests and meet regularly	Online technologies and tech support are provided and facilitated to regularly host social support networks online.	Available to older adults	• Self-referral	Free	Facilitated by a doctoral student in clinical psychology or psychology staff member under supervision of RRMC neuropsychologist.
* **Local Support Group**	Other surroundi						Identified during Focus Groups. No

**Provided by:**	ng community<br><br>**Community-based**						online information can be found, beyond contact information
**Respite Care<br><br>Provided by: Saskatchewan Health Authority: Sun Country Health Region (aka SCHR, Sun Country Health Region**	Coverage Area: Sun Country Health Region<br><br>Among others, includes:<br>• Community 1<br>• Community 4<br>• Community 2<br>• Community 3a<br>• Community 3b<br>• Other surrounding community<br><br>**Community-based**	**CAREGIVER RELIEF/RESPITE** Relief programs give caregivers some time off to look after themselves. **RESPITE CARE** is a service provided to give relief to the family or other primary caregivers of a dependent person living at home. **ADULT DAY PROGRAMS** provide organized health and social services in health care facilities to people who live in their own home but can travel to a nearby facility to participate in the programs.	Respite Care service is offered to caregivers, usually for a maximum of 60 days. The temporary beds are located throughout Health Region, and at long term care and integrated health centres. Temporary multi-purpose beds are utilized for convalescent and palliative care clients as well, in the provision of continuum service delivery.<br><br>Community 2 Adult Day Programs: 9:30 to 4 (Tues/Wed/Thurs)	Client Care Manager assessment of needs	Home Care, self, family, friends, health agencies. Application Process: contact your local Health Centre	Respite: minimum long-term rate of $32.94 per day.<br><br>Adult Day Program: $9.10/day (Community 2). Community 2 will add-on Care-a-van charge if needed to end-of-month program invoice.	Adult Day Programs **o**n hold due to pandemic; currently does out-workings to connect with previous participants to help in any way (help get to appts, etc)<br><br>**Education/Training of Service Providers:** Registered Nurses LPNs? Certified Care Assistants? Other?

**Respite Care**<br><br>**Provided by: Crocus Plains Villa Inc. Private Personal Care Home**	South East Saskatchewan (located in Community 2)<br><br>**Community-based**	Making a difference by promoting healthy senior living and providing quality of care through empowerment, innovation, and education.	Privately-run and operated home that provides lodging, meals, and assistance with or supervision of daily living. Authorized capacity for 30 residents.<br>**Includes respite care**, single rooms, wheelchair-accessible van.	Older adults. Care Level 1 to 4	Contact for information.	$1800 to $2199 (approximate monthly starting rate)	**Education/Training of Service Providers:** Full-time on-site nursing by Registered Nurse and Licensed Practical Nurse<br>Other?
****Home Care**<br><br>**Provided by: Saskatchewan Health Authority: Sun Country Health Region (aka SCHR, Sun Country Health Region)**	Coverage Area: Former Sun Country Health Region<br><br>Among others, includes:<br>• Community 1<br>• Community 4<br>• Community 2<br>• Community 3a<br>• Community 3b<br>• Other surrounding community	To help people who need assistance due to health problems and/or disabilities remain at home.<br>Goals:<br>- To teach self care and strengthen abilities.<br>- To delay/prevent loss of abilities.<br>- To assist families and other supporters.<br>- To provide referral service to other agencies.	Based on assessed need, the Region offers services from Registered Nurses, Home Health Aides, Case Managers, Volunteer Coordinators and Palliative Care Coordinators.<br><br>Services include:<br>• Assessment<br>• Homemaking<br>• Personal Care<br>• **Respite Care**<br>• **Meals on Wheels**<br>• **Nursing**<br>• Volunteers<br>• Palliative Care<br>• Equipment<br>Clients may be able to rent some special needs equipment for short periods of time while waiting for their own to arrive (such as walkers,	Based on assessed need, Saskatchewan residents who need acute, palliative and supportive care to remain independent at home. Out-of-province visitors have access through special arrangements.	Self, physician or NP, family member or friend. Certain nursing services require a physician or NP request. Contact the appropriate Home Care office.	Fees established by the Government of Saskatchewan which are government-subsidized for the patient and do not reflect the true cost of providing the service.<br><br>Home Care nursing, assessment and coordination services are free of	Community 1/Community 4 focus group identified a meal delivery program (possibly in Community 1, Other surrounding community) that was not Meals on Wheels, however no information can be found online.<br><br>**Meals on Wheels** is noted as a service offered through Home Care, where nutritionally balanced meals are delivered to the client's home, with the cost of the meal to the client is set by the Ministry of Health. As with other Home Care services, an application is made

	**Community-based**		wheelchairs, or bath benches).			charge to the client.	for service and an assessment is done.<br><br>**Education/Training of Service Providers:** Home Care Nurses Other?
***Home Care**<br><br>**Provided by: Comforts of Home Senior Care**	Community 2<br><br>**Community-based**	Providing in-home service to seniors, to assist them in remaining at home as long as possible.	Private, non-medical, in-home care for seniors; services include, but is not limited to, personal care (assisting with bathing, dressing, exercising), meals and nutrition, general household duties ranging from emptying trash and sorting recyclables, washing and folding laundry and light household cleaning. Also can help with grocery shopping, running errands, transportation to appointments or for social visits with friends and family, as well as provide companionship and respite care.	Older adults.	Self, other	No cost information identified.	**Education/Training of Service Providers:** Not identified
****Community 1 and District Handi-Van**<br>**Provided by: Town of Community 1**	Community 1 and district<br><br>**Community-based**	To provide transportation for the elderly and those with special needs.	Provides flexibly scheduled and routed transportation services using low-capacity vehicles for community residents who do not have private	Seniors and individuals requiring mobility assistance.	Self, other	$3 per round trip. Punch passes (good for 10 rides) are	**Education/Training of Service Providers:** None identified (volunteer-based)

			transportation and are unable to use the fixed-route public transportation system. Offered to seniors and mobility-impaired individuals.<br><br>Call to book a ride.<br><br>Hours of Operation: Wednesdays & Fridays 9:00 am - 4:00 pm Closed statutory holidays.			available for $30.	
***Volunteer Driver Program**	Services Community 4, Community 1, Other surrounding community<br><br>**Community-based**		Those with driver's licenses volunteer to help those who cannot drive				Identified in Focus Groups. Cannot find any other information online.
***Community 4 Handi-Van Provided by: Town of Community 4**	Community 4<br><br>**Community-based**	To provide transportation for the elderly and those with special needs.	Call to book a ride.<br><br>Hours of Operation: Mondays 11 am to 4 pm Thursdays 9 am to 1 pm Fridays 1 pm to 4 pm	Seniors and individuals requiring mobility assistance.	Self, other	$2 per round trip.	**Education/Training of Service Providers:** None identified (volunteer-based)
**** Handi-Van Provided by: Other surrounding community**	Other surrounding	Programs that provide flexibly scheduled and routed transportation services using low-	Hours of Operation: As needed<br><br>Call to book a ride.	Seniors and individuals requiring	Self, other	Service - $1/trip; $25 for distance of	2-1-1 lists this service but cannot be found on website provided

	communit y<br><br>**Communi ty-based**	capacity vehicles such as vans for community residents who do not have private transportation and are unable to use the fixed-route public transportation system.		mobility assistance.		less than 50km; $25 plus $.75/km for distances over 50km.	**Education/Training of Service Providers:** None identified (volunteer-based)
**Community 3b Handi-Van Provided by: Town of Community 3b**	Communit y 3b and within a radius of 50 kilometers .<br><br>**Communi ty-based**	Programs that provide flexibly scheduled and routed transportation services using low-capacity vehicles such as vans for community residents who do not have private transportation and are unable to use the fixed-route public transportation system.	Hours of Operation: 24/7 hours<br><br>Call to book a ride.<br><br>Those who wish to drive their family members in the Handi Van must first present their driver's licence to the Town Administrator. They must also take instructions from the Community 3b Health Centre on how to drive the van before using it.	Seniors and individuals requiring mobility assistance.	Self, other	Service - $3 one way. $6 return in town or $0.65 per kilometer out of town.	**Education/Training of Service Providers:** None identified (volunteer-based)
****Community 3a Handicap Bus Provided by: Town of Community 3a**	Communit y 3a and area<br><br>**Communi ty-based**	Programs that provide flexibly scheduled and routed transportation services using low-capacity vehicles such as vans for community residents who do not have private transportation and are unable to use the fixed-route public transportation system.	Call to book a ride.<br>Hours of Operation: 24/7 hours	Residents of Community 3a Marian Health Centre.	Self, other	Contact bus service for rates.	**Education/Training of Service Providers:** None identified (volunteer-based) Only staff of the Community 3a Marian Health Centre who have a valid driver's licence can operate the bus.<br><br>2-1-1 lists this service but no website provided and cannot

							be found on Town of Community 3a website and is not listed on SCHR Community 3a Marion Health Center website
****Care-a-van Provided by: Community 2 Care-A-Van Society**	Community 2 and surrounding area<br><br>**Community-based**	To provide transportation for persons of all ages who have mobility/disability problems	A van and a bus (both wheelchair accessible) available to rent, with or without a driver.<br><br>These vehicles are available for doctor appointments, church services, concerts, reunions, weddings, outings and other events.<br><br>Bus availability:<br>Monday - Friday 8:30 am - 4:30 pm. After hours available by request.<br>Van availability:<br>Weekdays and weekends. Book well in advance to ensure availability.	Persons of all ages who experience mobility or disability problems.	No referral process identified	Community 2: $10/person per trip ($20 round trip); anyone with class 5 licence can drive/rent – driver available for additional $20/hr.<br><br>2-1-1 also states:<br>Bus Rates<br>• $8 per ride each way<br>• After hours rates (in town) - $40 per hour (includes driver)<br>• After hour	Town helps support the van<br><br>**Education/Training of Service Providers:** None identified (volunteer-based)

						rates (out of town) - $40 per hour plus $0.60 per kilometer (includes driver)<br>Van Rates<br>• Daily rental $110 (includes 100 kilometers free, additional kilometers at $0.60 per kilometer<br>• Weekend rate (Friday evening until Monday morning) - $350 (includes 250 kilometers free, additional kilometers at $0.60 per kilometer	

						• In town rental - $30 for first hour or any part thereof. $20 per hour every hour after initial hour. Maximum of 60 kilometers for in town trips, above that a charge of $0.60 per kilometer will apply.	
**Learning Series:**<br>• **First Steps**<br>• **Next Steps for Families**<br>• **Care Essentials**<br>• **Options for Care**<br>• **Care in the Later Stages**<br>**Provided by: ASOS**	Classes are offered in two major cities, and throughout the province via Telehealth.	"Learn More Live Well"<br><br>The Learning Series helps people with dementia, their families and friends to live as well as possible with the disease.<br><br>A learning series for family members and	Participants will:<br>• Increase their knowledge of Alzheimer's disease and related dementia<br>• Discuss changes in communication<br>• Identify strategies to help cope with changes as they occur	People living with Alzheimer's disease and other dementias, family caregivers, and health care professionals	Self, other	All classes are offered free of charge, but donations to the Alzheimer Society of Saskatchewan are appreciate	**Education/Training of Service Providers:**<br>None identified

	All programming is currently virtual due to the pandemic.<br><br>**Provincial**	friends caring for individuals with Alzheimer's disease or a related dementia which provides an overview of the disease.	• Review relevant legal and financial issues<br>• Discuss local resources and support systems.<br>• Increase their knowledge about the progression of the disease, changes in communication, and responsive behaviours<br>• Learn more about practical approaches to day-to-day care<br>• Discuss strategies for reducing caregiver stress<br>• Identify available community resources.<br>• Increase their knowledge of changes that may occur with the progression of the disease<br>• Learn the signs of caregiver stress and coping strategies<br>• Review care and housing options in the community and through the Saskatchewan Health Authority<br>• Make plans to help with the transitions.<br>• Learn more about the later stages of Alzheimer's disease and other dementias			d and registration is usually required.	

			• Identify ways to provide care that promotes comfort, dignity and quality of life<br>• Understand loss and grief.				
****Public Awareness and Education Provided by: ASOS**	Province-wide<br><br>All programming is currently virtual due to the pandemic.<br><br>**Provincial**	"Learn More Live Well"<br><br>Educates the public about Alzheimer's disease and other dementias, and ways to lower the risk of developing the disease in various ways.	Offers community presentations such as **ABCs of Dementia**, providing information about the warning signs and getting a diagnosis; and **Heads Up for Healthier Brains**, which educates the public about brain health and dementia; and **Early Diagnosis – Getting a Diagnosis Toolkit** can help individuals to plan ahead to get the most out of their medical appointment and discuss their concerns with their family doctor or health care provider; and **Dementia Friends and Dementia Friendly Communities** Building a more inclusive Saskatchewan. Helps reduce the stigma and improve the quality of life of those affected by dementia through campaigns which inform the public about the scale and nature of dementia	Open to public. Registration is usually required.	Self, other	Free	**Education/Training of Service Providers:** None identified

			and empower individuals to seek a timely and early diagnosis.				
****An Evening of Education via Telehealth Provided by: ASOS**	Province-wide via Telehealth<br><br>**Provincial**	"Learn More Live Well"<br><br>Educates the public about Alzheimer's disease and other dementias, and ways to lower the risk of developing the disease in various ways.	An Evening of Education sessions are intended for family members and friends of people with dementia. Sessions offer hope by providing strategies and approaches to caregiving based on knowledge of dementia and its effect on the person.<br><br>Topics include the following as well as other topics:<br>• Person-centred communication strategies<br>• Strategies for activities of daily living, including meaningful activities<br>• Understanding loss and grief<br>• Using person-centred care principles for responsive behaviours	Open to public.	Self, other Registration required.	Free	**Education/Training of Service Providers:** None identified
****Alzheimer's Disease and Other Dementias - Information and Resources Provided by: ASOS**	Province-wide open online access<br><br>**Provincial**	"Learn More Live Well"<br><br>Educates the public about Alzheimer's disease and other dementias, and ways to lower the risk of	Visit the website to access information and resources for people with dementia, family members, caregivers and health professionals.<br><br>Includes:	Open to public.	Self, other	Free	**Education/Training of Service Providers:** None identified

		developing the disease in various ways.	• Extensive library of brochures and publications and videos<br>• Resources for health-care professionals.				
***Community 1 Active Living Program<br>Provided by: ??**	Community 1<br><br>**Community-based**	Aim to maintain or improve physical strength, mental capacity, and social skills of people living with dementia (thus far, those who have attended a Rural Memory Clinic)	Provides a structured two-hour session including 30 minutes of exercise, 30 minutes of brain stimulation games and an hour activity using fine and gross motor skills.	As yet, available to people living with dementia who have attended a rural memory clinic	Rural Memory Clinic team members	Free	Developed as a direct result of rural PHC memory clinics;<br><br>**Education/Training of Service Providers:**
****Minds in Motion<br>Provided by: ASOS**	Province-wide<br><br>Currently being offered virtually due to the pandemic.<br><br>**Provincial**	"Learn More Live Well"<br><br>Enjoy physical activity conducted by a certified fitness instructor, followed by activities and social time in a relaxed atmosphere. Develop new friendships with others living similar experiences.<br><br>**Benefits for the person living with dementia**<br>• Increased confidence and comfort with their diagnosis,<br>• Inclusion in community and<br>• Improved balance, mobility, flexibility and alertness.	A fitness and social program, in a relaxed atmosphere, for those with early stage memory loss and a friend, family member or caregiver.<br><br>Two hour, weekly program running for eight consecutive weeks.<br><br>The first part of the session will be a gentle physical fitness lead by a certified fitness instructor, the second part will be for a social activity time lead by Alzheimer Society volunteers.	Those with early stage memory loss and a friend, family member or caregiver.	Self, other	$30 per person	**Education/Training of Service Providers:**<br>Certified fitness instructor for physical activity portion; ASOS program staff &/or volunteers for social activity portion

		**Benefits for the caregiver, family member or friend**<br>• Self-care: An opportunity to focus on their own health, rather than focusing exclusively on the needs of the person living with dementia,<br>• Pleasure from seeing the person that they care for enjoying themselves and<br>• Mutual support and learning from other care partners.<br>**Benefits for all**<br>• Sharpened mental functioning, sometimes lasting two to three days,<br>• Reduced sense of isolation,<br>• Improved balance, mobility, flexibility, strength and endurance and<br>• Supportive environments which encourage new friendships with others who are living the same.					
****Coffee Clubs Provided by: ASOS**	Province-wide	"Learn More Live Well"	Provides an informal opportunity to socialize, share experiences, laugh,	People with dementia	Self, other	Free	**Education/Training of Service Providers:** None identified

	Currently being offered virtually due to the pandemic.<br><br>**Provincial**	To socialize, share and connect with others living with dementia.	and enjoy the company of others who understand and are living the dementia experience.	and/or their care partners.			
**Coffee and Chat Provided by: ASOS**	Province-wide<br><br>Currently being offered virtually due to the pandemic.<br><br>**Provincial**	"Learn More Live Well"<br><br>An informal way to check in with each other and ASOS staff.	Not a support group *(will not be able to address personal client matters during the chat)* but an opportunity to check in with each other and join some of the Alzheimer Society of Saskatchewan staff and peers online to say 'hello' and let us know how you are doing. We will be online via Zoom each Friday from 10:30-11:30 am.	People with dementia and/or their care partners.	Self, other	Free	**Education/Training of Service Providers:** None identified
****Community 1 Friendship Club**	Community 1<br><br>**Community-based**	No objectives identified.	Provides a variety of activities and events for older adults including fitness classes.	Older adults	Self, other	Membership - $10 per year	**Education/Training of Service Providers:** None identified
**Friendship Club Inc**	Other surrounding community<br><br>**Community-based**	No objectives identified.	Provides a variety of activities and events for older adults including pool, exercise classes, crafts, bridge, whist, shanghai, and smear.<br><br>Hours of Operation:<br>Coffee: everyday 9:30 am	Older adults	Self, other	Membership - $10 per year	**Education/Training of Service Providers:** None identified

			-11 am, 1 pm -3:30 pm<br>Bingo: last Thursday each month 7:30 pm<br>Cards: Tuesday night and Wednesday afternoon				
***Community 4 Happy Gang Club**	Communit y 4<br><br>**Communi ty-based**	A senior (55+) group that gets together to have FUN.	Education sessions, activities	Adults age 55+	Self, other	Membershi p $?	**Education/Training of Service Providers:** None identified
**Community 3a Senior Citizen Club**	Communit y 3a<br><br>**Communi ty-based**	No objectives identified.	Provides a variety of activities and events for older adults	Older adults	Self, other	Membershi p - $5 per year	Listed on Town of Community 3a site as a club/organization but no additional information<br>**Education/Training of Service Providers:** None identified
*** Seniors Drop-In Centre**	Other surroundi ng communit y<br><br>**Communi ty-based**	To encourage retired persons to participate in community life.<br>The Centre provides a place for any person fifty or over to become a member upon request for Membership.	Various activities, exercises, socials.<br><br>The Centre is to provide a place for social and cultural entertainment and for holding meetings. It aids in building friendships and encourages mutual aid among retired persons and others in the community.<br><br>Month regular meeting.	Adults age 50+	Self, other	Annual membershi p fees (actual cost cannot be found). Activity costs vary depending on activity.	Currently closed due to pandemic.<br>**Education/Training of Service Providers:** None identified
**Community 3b Drop-in**	Communit y 3b	No objectives identified.	Provides a variety of activities and events for older adults including:	Older adults	Self, other	Membershi p - $10 per year	**Education/Training of Service Providers:** None identified

	**Communi ty-based**		cards, bingo, floor shuffleboard, and pool.<br><br>Monday-Saturday, 2:30 pm to 5 pm				
**Wheatland Senior Centre**	Communit y 2<br><br>**Communi ty-based**	No objectives identified.	Provides a variety of activities and events for older adults.<br>Hours of Operation: Office Hours: Monday - Friday 9 am - noon Contact the office for information about various activities.	Older adults	Self, other	Membershi p - $25 per year or $45 for two years	**Education/Training of Service Providers:** None identified
* **Over Sixty Club**	Other surroundi ng communit y<br><br>**Communi ty-based**						Identified during Focus Groups. No online information can be found, beyond contact information<br><br>**Education/Training of Service Providers:** None identified
Four Seasons Drop-In Centre	Other surroundi ng communit y<br>**Communi ty-based**		Activities, games, exercises, special Tues just for seniors				Identified during Focus Groups.<br><br>**Education/Training of Service Providers:** None identified
**Southeast Regional Public Libraries**<br><br>**Outreach program was identified in focus group;**	Multiple locations in Southeast Saskatche wan including	Our Values:<br>• Effective literacy skills<br>• Free and uncensored access to information<br>• Accessible collection resources	Events, reading club, crafts, various activities<br><br>**Community 2**<br>**"Outreach Program"** - **home book delivery** for	Some programs for Adults (ages 18+); Seniors (Outreach)	No referral; library card & registration required.	No informatio n re: cost identified	**Education/Training of Service Providers:** None identified

**others not explicitly identified**	Community 1, Community 2, Community 3a, Community 3b, Community 4, Other surrounding community<br><br>During pandemic some services are now offered virtually<br><br>**Community-based**	• Innovation and creativity in life<br>• Informed personal decision making<br>• Freedom and individual privacy<br>• Participation in society<br>• A central place in the community<br>• Informed and capable staff<br>• Equitable access to services<br>• Community oversight and trusteeship<br><br>Our collection includes free information and kits you can't get anywhere else:<br>• Books for all ages, including large print<br>• DVDs and Blu-Rays<br>• Audiobooks on CD<br>• e-Books and e-Audiobooks<br>• Video Games (Wii, Wii U, Xbox 360, Xbox One, PlayStation 4)<br>• Makerspace Kits (in-branch use only)<br>• Book Club in a Bag Kits<br>• Literacy Kits (in-branch use only)	'seniors or adults' unable to get to library.				

		• Magazines and newspapers (physical and digital)<br>• NNELS for public library patrons who cannot read traditional print<br>Full suite of online databases including health info, auto repair manuals, craft tutorials, and much more.					
**MedicAlert® Safely Home®**<br>**Provided by: MedicAlert® Safely Home®**<br>**in partnership with Alzheimer Society of Canada**	Canada-wide<br><br>**NATIONAL**	MedicAlert Safely Home helps ensure that people living with Alzheimer's disease or dementia are quickly reunited with their families. This nation-wide program in partnership with the Alzheimer Society of Canada offers eligible subscribers a unique MedicAlert blue bracelet for people with dementia for free with their service plan.	MedicAlert® Safely Home®, in partnership with MedicAlert® Foundation Canada, is a medical identification service for people with Alzheimer's disease or a related dementia. Subscribers receive an identification bracelet engraved with critical information and the 24/7 Emergency Hotline number, and this information can be used to quickly reunite people with dementia with family if they go missing or become lost in the community.<br>• 24/7 Emergency Hotline<br>• MedicAlert® Identification	Persons with Alzheimer's disease or a related dementia.	Self, other	$60 for 1 year of MedicAlert protection.	**Education/Training of Service Providers:** None identified

			• Family Notification				
