# Supplementary material for: Services for older adults in rural primary care memory clinic communities and surrounding areas: a qualitative descriptive study
Source: BMC Health Serv Res. 2024 Jun 13;24:725. doi: 10.1186/s12913-024-11167-w (PMC11170901; doi:10.1186/s12913-024-11167-w)
Supplement: Supplementary file 3 — Supplementary Material 3: Additional File 3. Caregiver Interview Guide. This semi-structured interview guide was developed based on Phase 1 findings, and used for each of the five caregiver interviews. [file 12913_2024_11167_MOESM3_ESM.pdf]

Environmental Scan of Community Programs
Caregiver Interview Guide

ID: ____________

Date: ____________

Introduction:

We are conducting an Environmental Scan about local community programs and services in your area that might be used by people living with dementia and family caregivers**.**

The goal of this interview is to help us better understand the experience of people with dementia and their family caregivers who use these local community programs and services.

Demographics

1. For the purposes of this interview, would you describe yourself as a person living with dementia or a family member of a person living with dementia? *(spouse/partner/child/other?)*

2. What year were you born?

3. What sex were you assigned at birth, on your original birth certificate *(Male/Female)* **and does your sex also best describe your current gender identity?** *(Male/Female/Other [for example, non-binary])*

4. What is your ethnic background? *(First Nations/Metis/Inuit/European/Asian/African/Other)*

5. What is your current living arrangement? *(Do you live alone? With spouse? With other family member?)*

6. Did you attend a rural primary health care memory clinic and if so, which clinic did you attend?

7. Have you received a diagnosis related to dementia and if so, what diagnosis did you receive? *(MCI, AD, FTD, VD, etc)*

8. Would you say that you live *in* a city, town, or village or *outside of* a city, town or village *(for example, on a farm or acreage)***?**

- *[If on a farm or acreage]* **How far do you live from the closest city, town, or village?**
- **Would you happen to know the approximate population?** *[of the city/town/village in which you live, or that you live nearest to]*

Community Programs and Services

1. I am going to list and describe seven types of local community-based programs and services for older adults and as we go through each one, I will ask you a few questions such as, are you aware of the service and if you have used it in the last few years, etc.

A. **Are you aware of any programs and services that offer** *"various programs and services will be listed/described here"*
    - **If yes:**
        i. **Do you recall how you became aware of these services?** *(health provider, friend, advertisement, other)*
    - **If no:**

i. **Do you think this type of service might be helpful to you?** *(explore, why/why not; then begin again with next service type)*

B. Have you used this type of service in the last few years, say since 2019? *(one year before pandemic started, to present)*

- **If yes:**
  i. **Why did you decide to use this service?** *(explore needs)*
  ii. **If you participated in a group setting, were there both males and females present and did you feel that the topic or intent of the service was of equal interest to both?** *(explore)*
  iii. **Do you recall if the service provider(s) was male or female and whether that impacted your experience?** *(explore)*
  iv. **Do you feel that the service was helpful? How so?** *(why/why not; best/worst)*
  v. **Would you have any recommendations that might improve this service?** *(explore)*
  vi. **Did you have any difficulty accessing or using this service? How so?** *(for example, cost/internet/ transportation/other)*
    - **Do you feel there might be ways to work around those difficulties that would be helpful?** *(explore)*
  vii. **Is there anything about this services that made it easier for you to access and use?**
- **If no:**
  i. **Can you tell me about why you have not used these services***? (for example, experienced barriers such as travel, cost, no internet, not of interest or not needed, not available during pandemic, other)* **Do you feel there might be ways to work around those barriers that would be helpful?**

2. Are you aware of any other programs/services that might be beneficial to you but that you have not used and that we haven't already discussed? *(explore: how so/why not used)*

3. Do you feel like the pandemic has had an impact on *your* use of services and programs and how so? *(explore: negative or positive impact of for example, changes in availability, accessibility, new programs, or on your planned use of services and programs in the future)*

4. If you had the opportunity to spend one minute talking to an influential decision-maker about community-based programs and services in your area, what might you say?

5. Is there anything else you would like to share that we haven't already talked about today or do you have any questions?

Thank you so much for participating today. We really appreciate your time and value your input.
